# Oxyresveratrol attenuates bone resorption by inhibiting the mitogen-activated protein kinase pathway in ovariectomized rats

**DOI:** 10.1186/s12986-024-00781-4

**Published:** 2024-01-19

**Authors:** Yea-Jin Lee, Jin-Chul Ahn, Chung-Hun Oh

**Affiliations:** 1 Department of Medicine, Dankook University, Cheonan-si, 31116 Republic of Korea; 2 Medical Laser Research Center, Graduate School of Medicine, Dankook University, Cheonan-si, 31116 Republic of Korea; 3 Department of Photobiology, College of Medicine, Dankook University, Cheonan-si, 31116 Republic of Korea; 4 Department of Oral Physiology, College of Dentistry, Dankook University, Cheonan-si, 31116 Republic of Korea

**Keywords:** Mitogen-activated protein kinase, Osteoclast, Osteoporosis, Ovariectomized rat, Oxyresveratrol

## Abstract

**Background:**

Bone is continuously produced by osteoblasts and resorbed by osteoclasts to maintain homeostasis. Impaired bone resorption by osteoclasts causes bone diseases such as osteoporosis and arthritis. Most pharmacological treatment of osteoporosis focuses on inhibiting osteoclast differentiation, often to restore osteoclast/osteoclast balance. However, recent osteoporosis treatments have various side effects. According to a recent study, resveratrol, known as a stilbenoid family, is known to increase bone density, and the osteoclast inhibitory effect was confirmed using oxyresveratrol, a stilbenoid family. Here, we investigated the effect of oxyresveratrol on osteoclast differentiation and an ovariectomized mouse model.

**Methods:**

Mouse leukemia monocyte/macrophage cell line RAW 264.7 was treated with oxyresveratrol, and cell cytotoxicity was confirmed by measuring MTT assay. Tartrate-resistant acid phosphatase (TRAP), an enzyme marker for osteoclasts, was confirmed by staining. In addition, osteoclast differentiation markers and MAPK-related markers were confirmed at the mRNA level and protein expression. The effect of oxyresveratrol was confirmed using ovariectomized mice. Deoxypyridinoline (DPD) was measured using mouse urine and TRAP activity was observed using serum. Bone mineral density was also measured using Micro-CT.

**Results:**

The polyphenol oxyresveratrol inhibited receptor activator of nuclear factor kappa-Β ligand (RANKL)-induced osteoclast differentiation of RAW 264.7 cells. Furthermore, oxyresveratrol inhibited TRAP activity and actin-ring formation. Moreover, oxyresveratrol suppressed the phosphorylation of the RANKL-induced mitogen-activated protein kinases (MAPKs) p38, JNK, and ERK and significantly reduced the expression of bone differentiation markers (NFATc1, cathepsin K, and TRAP).

**Conclusion:**

Oxyresveratrol inhibits osteoclast differentiation via MAPK and increases bone density in ovariectomized rats, suggesting it has therapeutic potential for bone diseases such as osteoporosis. We confirmed the osteoporosis prevention effect of OR in Raw 264.7 cells, and future studies should confirm the effect of OR using rat bone marrow-derived cells.

## Background

Bones form the body structure, protect organs, and play a crucial role in facilitating muscle actions [1]. Bones are typically generated by osteoblasts and resorbed by osteoclasts, maintaining a homeostatic balance, and bone diseases result when this balance is disrupted [2]. Osteoclasts originate from the monocyte/macrophage lineage, and a rapid increase in osteoclast differentiation leads to bone diseases such as osteoporosis [3]. Pharmacologic treatment of osteoporosis often focuses on inhibiting the differentiation of osteoclasts to restore the osteoblast/osteoclast balance. However, such treatments have multiple side effects. For example, bisphosphonates, the most widely used group of drugs for osteoporosis, carry risks such as mandibular fracture; femoral fracture; and severe necrosis of muscles, joints, or bones [4]. Multiple factors are implicated in osteoclast differentiation. Although the steps involved in the differentiation of monocytes/macrophages, which in turn influence osteoclast differentiation, are unknown, receptor activator of nuclear factor-kappa B (NF-κB) ligand (RANKL) and macrophage colony-stimulating factor (M-CSF), which are expressed in macrophage precursor cells, induce osteoclast differentiation [2, 5–7]. RANKL is a member of the tumor necrosis factor family [2, 6] that promotes osteoclast differentiation by recruiting other adapter molecules and activating NF-κB and mitogen-activated kinase (MAPK), resulting in the formation of multinucleated bone-resorbing osteoclasts [7–9]. MAPK consists of p38 protein kinase and c-Jun N-terminal kinase/stress-activated protein kinase (JNK/SAPK) as well as extracellular signal control key (ERK) 1/2, Tyr, and Ser/Thr protein kinases [10, 11]. Among the MAPKs, ERK, JNK, and p38 activate the transcription factor activator protein 1 [nuclear factor of activated T-cells cytoplasmic 1 (NFATc1)] which promotes osteoclast differentiation. NFATc1 subsequently translocates to the nucleus and promotes osteoclast differentiation via the actions of other transcription factors [12–17]. NFATc1 triggers osteoblast fusion and activation by upregulating genes related to osteoblast adhesion, migration, acidification, and decomposition of inorganic and organic bone substrates, such as tartrate-resistant acid phosphatase (TRAP), dendritic cell-specific transmembrane protein (DC-STAMP), and cathepsin K [18]. Stilbene has two aromatic rings connected by a double bond and is classified as either trans- or cis-stilbene. Cis-stilbene is less structurally stable than trans-stilbene, because the bonding between the two aromatic rings is hindered. Thus trans-stilbene is the more common form in nature. Stilbenoids, which are stilbenes with hydroxyl groups, are classified as polyphenols and are present in the red grape peel and red wine [19]. Resveratrol and oxyresveratrol (OR) are the most well-known stilbenoids [20–22]. OR has four hydroxyl groups and was discovered in *Artocarpus lacucha* (Moraceae) [19]. Since OR has more hydroxyl groups than resveratrol, it is considered more biologically active [19]. Compared to resveratrol, OR has 1.5–twofold higher antioxidant activity [23], lower cytotoxicity [24], and higher anti-inflammatory activity [25]. OR also demonstrates whitening [26, 27], anticancer [28, 29], and neuroprotective effects, since it is capable of crossing the blood–brain barrier, as reported in a rat model of stroke [30]. Resveratrol can reportedly prevent osteoporosis [31]. In a recent study, OR, possessing one more hydroxyl group than resveratrol, has demonstrated lower cytotoxicity and better antioxidant effects. However, the mechanism of OR on osteoclast differentiation remains unclear. Here, we evaluated the effect of OR on osteoporosis. Our findings demonstrated that OR inhibited osteoclast differentiation in a rat model in a MAPK-dependent manner.

## Methods

### Culture of RAW 264.7 cells

RAW 264.7 mouse monocytes were purchased from the American Type Culture Collection (ATCC #TIB-71; Manassas, VA, USA). To culture RAW 264.7 cells, 50 mL of fetal bovine serum (FBS; Welgene, Gyeongsan, Korea) and 100 units/mL penicillin–streptomycin (Welgene) were added to 500 mL of Dulbecco’s modified Eagle medium (DMEM; Welgene). Cells were cultured in an incubator (Thermo, Waltham, MA, USA) with 5% CO_2_ and 95% air at 37 °C. Cells were enumerated using a hemocytometer (Paul Marienfeld, Lauda-Königshofen, Germany), and their morphology was observed using an optical microscope (Olympus, Tokyo, Japan).

### Induction of osteoclast differentiation and OR treatment

Cells were plated at a concentration of 5 × 10^4^/mL in 96-well plates and maintained in DMEM containing 10% FBS. After 24 h, the medium was replaced with alpha-Minimum Essential Medium (alpha-MEM; Welgene), and cells were treated with 100 ng/mL RANKL (PeproTech, East Windsor, NJ, USA) and 1 Nm to 10 µM OR (Sigma-Aldrich, St. Louis, MO, USA) for 3 d to induce osteoclast differentiation.

### Cell cytotoxicity assay

Cells were seeded in a 96-well plate at 3 × 10^4^/mL, and treated with OR and RANKL (100 ng/mL) for 24 h. Next, 50 µL from each well was added to thiazolyl blue tetrazolium bromide (MTT; Sigma) in Dulbecco’s phosphate-buffered saline, and cultured for 2 h. The supernatant was removed, and 150 µL of dimethyl sulfoxide (Daejung, Busan, Korea) was added to dissolve formazan crystals. The absorbance at 540 nm was measured using a microplate reader (Biochrom, Cambridge, United Kingdom).

### TRAP activity assay

To assay TRAP activity, cells were seeded at 5 × 10^4^/mL in a 96-well plate in alpha-MEM with 50 ng/mL RANKL, and treated with OR for 3 days. Cells in 96-well plates were washed once with phosphate-buffered saline (PBS) and lysed in 80 µL of cold lysis buffer (90 mM citrate [Ph 4.8] and 0.1% Triton X-100 containing 80 mM sodium tartrate) for 10 min. Substrate solution (80 µL; 20 mM *p*-nitrophenylphosphate) was added, and the cells were incubated for 30 min at 37 °C. To quench the reaction, 40 µL of 0.5 N NaOH was added, and the optical density at 405 nm was determined.

### TRAP staining

Cells were seeded at 5 × 10^4^/mL in a 96-well plate in alpha-MEM with 50 ng/mL RANKL, and treated with OR for 3 days. Cells were washed once with PBS and fixed in 10% formalin for 5 min. After fixing, the cells were washed with distilled water three times. TRAP staining solution was prepared following the manufacturer’s instructions (acid phosphatase kit no. 387; Sigma). Stained cells were observed by optical microscopy (Olympus), and images were captured using the attached digital camera (DIXI 3000; Olympus). Round osteoclasts (ROCs) were manually enumerated.

### Real-time quantitative polymerase chain reaction

Total RNA was extracted from cells using TRIzol (Invitrogen-Gibco, Grand Island, NY, USA). cDNA was synthesized using the iScript™ cDNA Synthesis Kit (Bio-Rad, Hercules, CA, USA) according to the manufacturer’s instructions. Polymerase chain reaction (PCR) primers were from Bioneer (Oakland, CA, USA). SYBR Green-based real-time PCR was performed on a StepOnePlus™ Real-Time PCR System (Applied Biosystems, Foster City, CA, USA).

### Rhodamine phalloidin staining

Raw 264.7 cells were seeded at 1 × 10^5^/mL in a two-well plate (SPL, Pocheon, Korea) for 24 h. Next, RANKL (100 ng/mL) and OR were added for 3 days. After washing twice with PBS, the cells were fixed in 3.7% formaldehyde at 24 °C for 10 min. Following two washes with PBS, 0.1% Triton-X 100 was added at room temperature for 5 min. After washing with 1% bovine serum albumin (BSA; Santa Cruz Biotechnology, Dallas, TX, USA) at room temperature for 20–30 min, the cells were washed with PBS. Rhodamine phalloidin (200 µL; Life Technologies, Carlsbad, CA, USA) was added to each well. After treatment with 300 µL of 4’,6-diamidino-2-phenylindole (DAPI; Sigma) at room temperature for 5 min, actin rings were observed by fluorescence microscopy (IX53; Olympus).

### Western blotting

RAW 264.7 cells were seeded in 100-mm dishes at 1 × 10^6^/mL and cultured in DMEM containing 10% FBS for 24 h. OR-treated cells were lysed in RIPA buffer with protease inhibitor cocktail (Sigma). Cell lysates were centrifuged at 16,128*g* for 20 min. Protein samples (30 µg) were resolved by sodium dodecyl sulfate–polyacrylamide gel electrophoresis (Bio-Rad) and transferred to a polyvinylidene fluoride membrane (Bio-Rad). The membrane was blocked using 5% skim milk (Bio-Rad), and antibodies against Phospho-ERK, ERK, Phospho-JNK, JNK, Phospho-p38, p38, and the osteoclast differentiation markers NFATc1, cathepsin K, and TRAP (Cell Signaling Technology, Danvers, MA, USA) in 1% BSA were added overnight at 4 °C. Rabbit anti-mouse IgGs were used as secondary antibodies for 1 h at room temperature. Signals were detected using the ChemiDoc Imaging System (Bio-Rad).

### Animals

Five-week-old female Sprague–Dawley rats were purchased from Nara Biotech (Seoul, Korea). All animal procedures adhered to the guidelines of the Institutional Animal Care and Use Committee of Dankook University (approval #DKU-18-040). Animals were housed at 24 ± 1 °C, with 60 ± 5% humidity, and a 12/12 h light and dark cycle (lights on at 9 am and off at 9 pm). The rats’ diet consisted of basic solid feed (Nara Biotech) and drinking water. Ovariectomy was performed at 6 weeks of age to induce osteoporosis. Rompun (Bayer, Germany) and Zoletil 50 (Virbac, USA) were mixed at a ratio of 1:4 and administered intraperitoneally at 1 mL/kg, followed by general anesthesia. Both abdominal hair were removed and sterilized with povidone-iodine (PVP-I; Green Pharmaceutical, Korea). The skin, muscle layer, and peritoneum were incised ~ 2 cm, the ovaries were exposed, the lower parts of which were tied with Biacryl silk (4–0, 19 mm, 3/8C, 45 cm; Ethicon, Raritan, NJ, USA), and the ovaries were excised. Next, the peritoneum, muscle layer, and skin were sequentially sutured with black silk (SK441, 4-0, 21 mm, 3/8C, 50 cm; Airi, Korea). The sham group underwent the same surgical procedure except for ovarian resection. To prevent infection after surgery, gentamicin (Komipharm, Siheung, Korea) was injected intramuscularly at 0.2 mg/kg. The osteoporosis induction period was 2 weeks after surgery, and 1 mL of the drug was administered orally daily for 8 weeks. The rats were divided into the sham group (sham), ovariectomy group (OVX), 200 µg/kg alendronate group (AND), and three OR groups at 1 mg/kg, 10 mg/kg, and 20 mg/kg (n = 5 each).

### Deoxypyridinoline assay

To assay deoxypyridinoline (DPD), rat urine was collected in the morning after fasting overnight. Urine samples were collected and stored at − 20 °C. We used rat DPD and an ELISA Kit (Elabscience, Houston, TX, USA) to assay DPD levels. To correct for dilution, results were normalized to urinary creatinine and expressed as nanomoles of DPD per millimole urinary creatinine.

### Measurement of TRAP activity in serum

TRAP activity in serum was measured using a kit (Takara, Kusatsu, Japan). Serum was diluted in dilution buffer, and 50 µL of substrate solution was added to each well and reacted at 37 °C for ≤ 60 min. Next, 50 µL of stop solution was added, and the absorbance at 405 nm was measured.

### Analysis of micro-CT

The femur was imaged by micro-CT using a three-dimensional microfocus computed tomography instrument (Sky-Scan 1176; Sky-Scan, Kontich, Belgium). The sample was wrapped in plastic wrap to prevent drying. X-ray alignment was performed by the software alignment system; the program setting was 2000 × 1336 (medium), the camera focus was set to 18 μm, and an aluminum filter of 1 mm was used. Images were reconstructed using NRecon software (ver. 1.6.9.4; Bruker, Billerica, MA, USA), and coronal, sagittal, and transverse bone structures were evaluated using DataViewer software (ver. 1.5.1.2; Bruker). Three-dimensional analysis was performed using CTAn software (ver. 1.13.5.1+; Bruker), and three-dimensional rendering was using CTvol software (ver. 2.2.3.0; Bruker). The femur was scanned using CT-analyzer™ software (SkyScan). All samples were analyzed with the same anatomical structure, and a thickness of 2 mm was set at a distance of 2 mm from the growth plate. The percentage bone volume to total volume (BV/TV) of the samples was calculated.

### Statistical analysis

Utilize GraphPad Prism 7.0 to analyze and visualize the experimental data. Results are expressed as mean ± standard error of the mean and were compared using a two-tailed Student’s *t*-test. A value of *p* < 0.05 was accepted as statistically significant. Results are for representative experiments performed in at least triplicate.

## Results

### Effect of OR on cell cytotoxicity

**Fig. 1:**
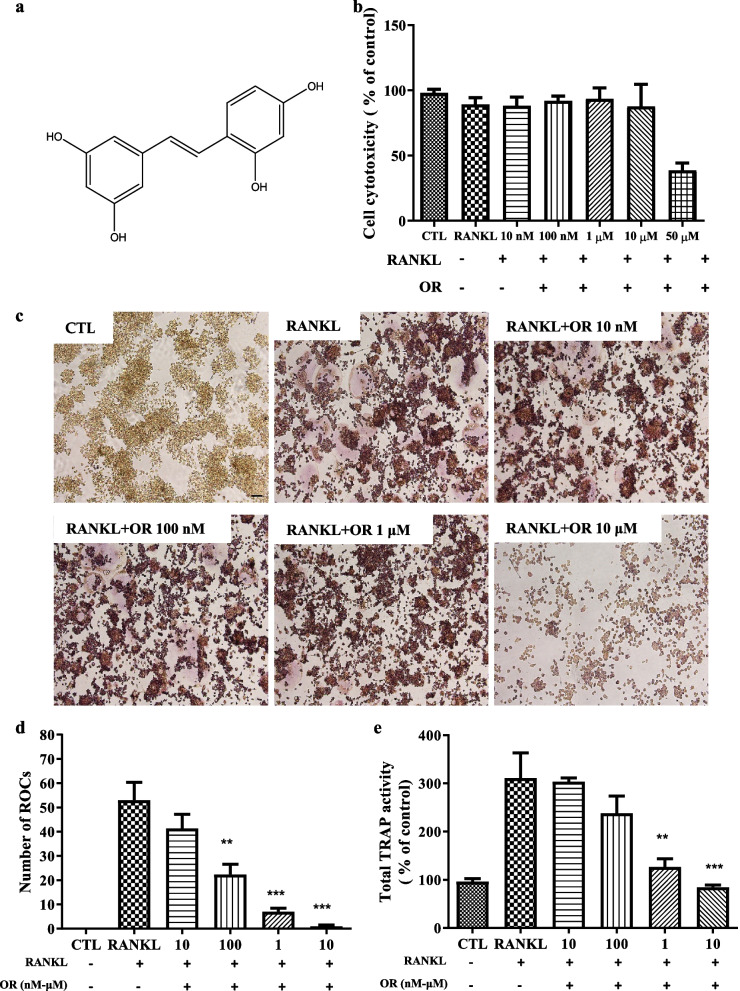
Inhibition of osteoclast differentiation by oxyresveratrol (OR) at non-cytotoxic concentrations. (**a**) Structure of OR. (**b**) RAW 264.7 cells were treated with OR for 3 days and subjected to MTT assay. Data represent percentages *versus* control (CTL). Ordinary one-way ANOVA; Dunnett’s multiple comparisons; n = 3; *** p < 0.001. (**c**) TRAP staining and the formation of TRAP-positive multinucleated RAW 264.7 cells. Cells were seeded at 5 × 10^4^/mL in a 96-well plate and cultured for 3 days in the presence of OR (10 nM, 100 nM, 1 μM, and 10 μM). RANKL induced the formation of TRAP-positive multinucleated RAW 264.7 cells. Original magnification, × 200. (**d**) ROC number of TRAP-positive multinucleated cells. (**e**) TRAP activity of TRAP-positive multinucleated cells. Data are percentages *versus* RANKL. **p < 0.01, ***p < 0.001, n = 3. Error bars, standard deviations. OR, oxyresveratrol; RANKL, receptor activator of nuclear factor kappa-Β ligand; TRAP, tartrate-resistant acid phosphatase; ROC, round osteoclast

The structure of OR is shown in Fig. 1a. Treatment with 50 μM OR and RANKL (100 ng/mL) for 3 days decreased the viability of RAW 264.7 cells to < 50%; other OR concentrations did not exert significant effects (Fig. 1b). Therefore, OR concentrations exceeding 50 μM were considered cytotoxic and excluded from subsequent experiments.

### Effect of OR on osteoclast differentiation

TRAP staining showed that RANKL promoted osteoclast differentiation, and ROC staining was not significantly different between 10 nM OR and RANKL alone. The number of ROCs was decreased by 100 nM, 1 μM, and 10 μM OR (Fig. 1c). OR at concentrations other than 10 nM suppressed the differentiation of ROCs to osteoclasts (Fig. 1d).

### Effect of OR on TRAP activation in osteoclasts

TRAP activity did not differ significantly between 10 nM OR and RANKL alone. Compared to RANKL alone, OR at 100 nM, 1 μM, and 10 μM decreased TRAP activity in a dose-dependent manner (Fig. 1e).

### Effect of OR on actin-ring formation and bone resorption

**Fig. 2:**
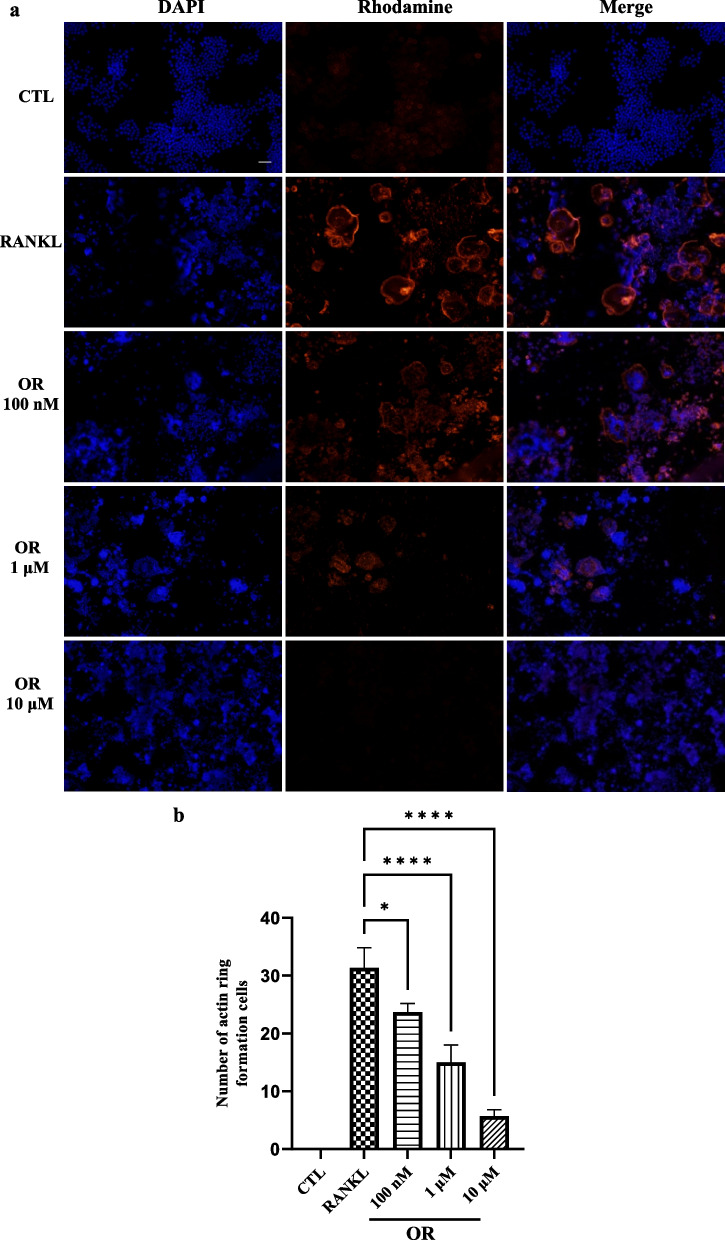
Effect of oxyresveratrol (OR) on actin ring formation in RAW 264.7 cells. (**a**) Osteoclasts were stained with rhodamine phalloidin to observe actin rings; nuclei were visualized with DAPI. RAW 264.7 cells were seeded onto two-well plates at a density of 1 × 10^5^/mL. (**b**) Quantitative analysis of the number of actin rings in RAW 264.7 cells. Error bars indicate standard deviation. Ordinary one-way ANOVA; Tukey’s test for multiple comparisons; n = 3; *p < 0.05; **** p < 0.0001. Original magnification, × 200

Actin rings, which create secure sealing zones, are morphological markers of bone resorption after osteoclast differentiation. Rhodamine phalloidin staining showed that ROCs treated with RANKL had pericellular actin rings. OR suppressed actin-ring formation in a dose-dependent manner (× 200). DAPI staining showed that 100 nM to 10 μM OR inhibited osteoclast differentiation (Fig. 2).

### MAPK protein levels in osteoclasts

**Fig. 3:**
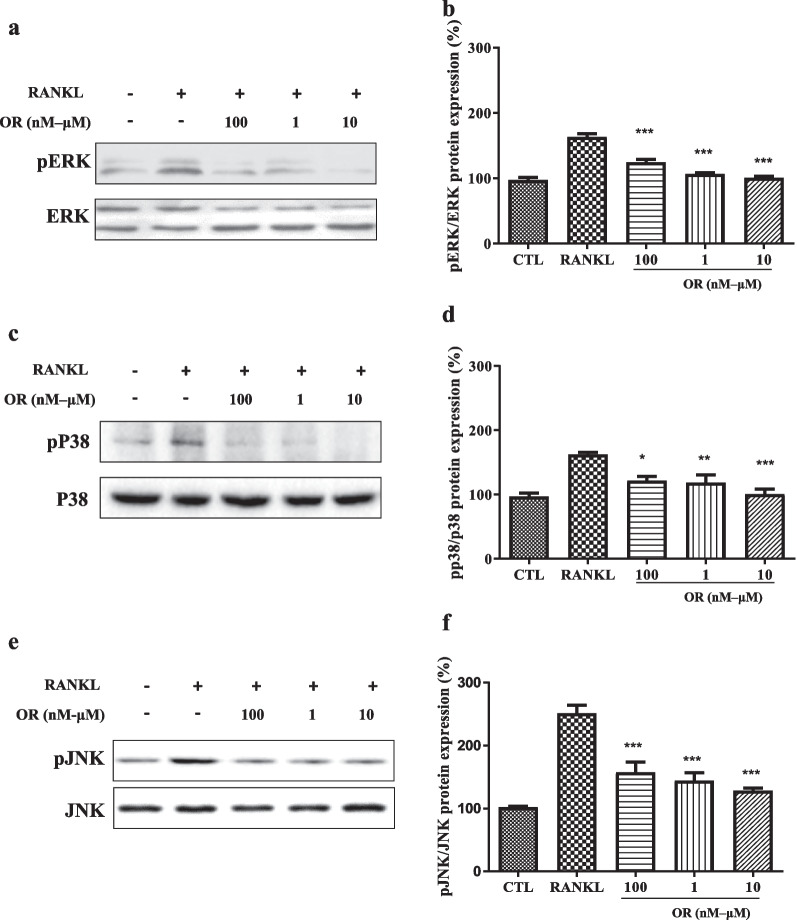
Effect of oxyresveratrol (OR)-mediated mitogen-activated protein kinase (MAPK) inhibition on RANKL-induced osteoclast differentiation. pERK, pJNK, and pP38 levels in RAW 264.7 cells by western blotting. Levels of (**a**) pERK, (**c**) pP38, and (**e**) pJNK after treatment with RANKL and OR (100 nM, 1 μM, and 10 μM). Cells were harvested after 15, 240, and 30 min for pERK, pP38, and pJNK, respectively. Protein levels of (**b**) pERK, (**d**) pJNK, and (**f**) pP38. ERK, P38, and JNK were used as loading controls. Data are percentages *versus* RANKL (***p < 0.001, n = 3)

MAPK/AP-1 pathways are important in osteoclastogenesis [32, 33]. Western blotting indicated that OR inhibited the RANKL-induced phosphorylation of ERK, p38, and JNK but did not affect the total ERK, p38, and JNK levels (Fig. 3a–f). Therefore, 100 nM to 10 μM OR inhibited RANKL-induced MAPK activity.

### Induction of marker genes of osteoclast differentiation by RANKL

**Fig. 4:**
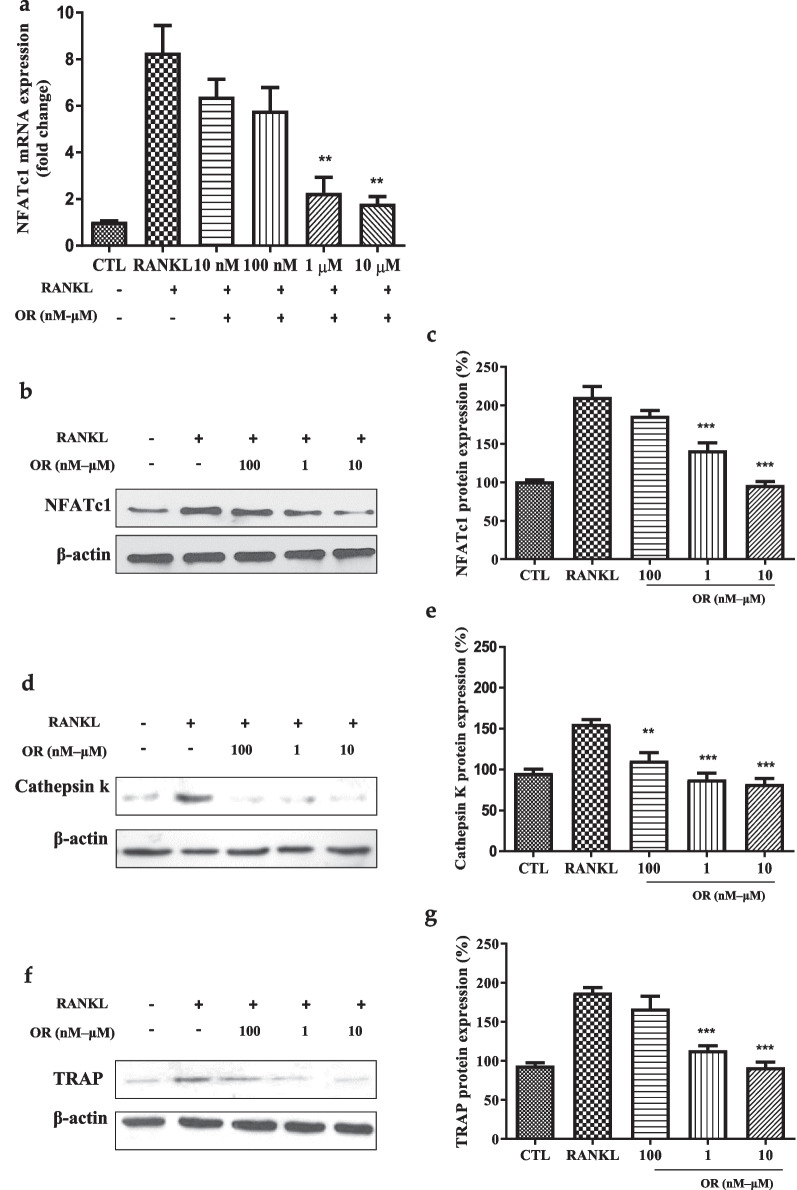
Effect of oxyresveratrol (OR) on RANKL-induced bone-differentiation and bone-resorption marker genes. (**a**) Effect of OR on the NFATc1 mRNA level during osteoclast differentiation of RAW 264.7 cells. RAW 264.7 cells were seeded onto 35-mm culture dishes at 2 × 10^6^/mL. Data are percentages *versus* RANKL (*p < 0.05, **p < 0.01, n = 3). (**b–g**) Effect of RANKL and OR (1 μM, or 10 μM) on NFATc1 (**b**, **c**), cathepsin K (**d**, **e**), and TRAP (**f**, **g**) protein levels in RAW 264.7 cells by western blotting. β-actin was used as the loading control. Data are percentages *versus* RANKL (*p < 0.05, ***p < 0.001, n = 3)

Osteoclast differentiation results in elevated expression of osteoclast marker genes. OR decreased the expression of NFATc1, an osteoclast differentiation marker, in a dose-dependent manner (Fig. 4a).

### Expression of osteoclast differentiation-associated marker proteins

OR at 100 nM, 1 μM, and 10 μM reduced the expression of NFATc1, suggesting suppression of bone differentiation (Fig. 4b, c) and cathepsin K and TRAP, suggesting suppression of RANKL-induced bone resorption (Fig. 4d–g).

### DPD and TRAP activity after ovariectomy

**Fig. 5:**
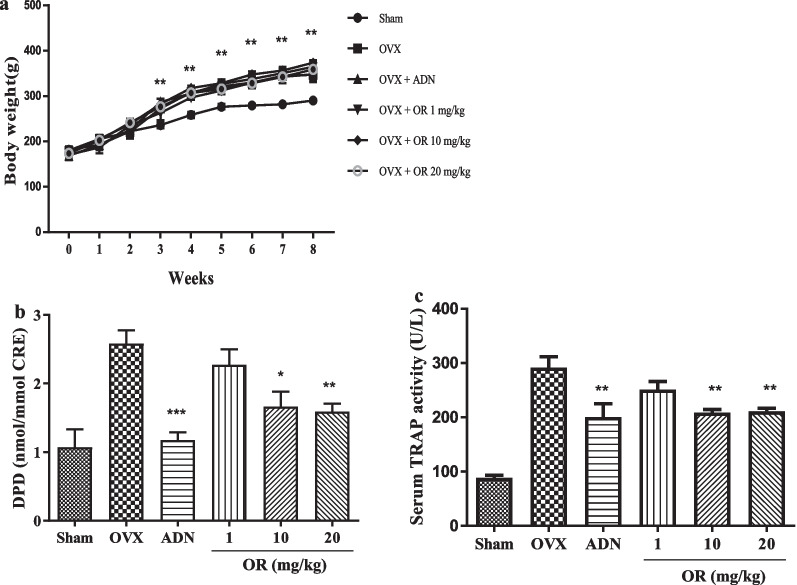
Effects of bone density in oxyresveratrol (OR) treatment on OVX mouse model. (**a**) The body weight of the rat was measured weekly after the start of the experiment. It was confirmed that the weight increased in all groups except the sham group. (**b**) After normalization to creatine in the group treated with 10 and 20 mg/kg of OR, DPD activity was decreased, but not as much as in the ADN group (n = 5); *p < 0.05, **p < 0.01, ***p < 0.001 compared with OVX group. Error bars, standard deviations. (**c**) Serum TRAP enzyme activity (mean ± SD) in rats in the OVX group was compared to the OR group. The decrease was confirmed in the OR groups of 10 and 20 mg/kg. (n = 5); **p < 0.01, ***p < 0.001 compared with OVX group. Error bars, standard deviations

After ovariectomy, the body weight of rats increased in all groups except the sham group. Weight increase was confirmed through OVX (Fig. 5a). Urinary excretion of deoxypyridinoline (DPD) was quantified as a marker for bone resorption. DPD activity in rat urine increased in the OVX group. OR at 10 and 20 mg/kg decreased DPD activity after normalization to creatine (Fig. 5b). TRAP activity in serum was significantly decreased by OR at 10 and 20 mg/kg (Fig. 5c).

### X-ray microtomography analysis

**Fig. 6:**
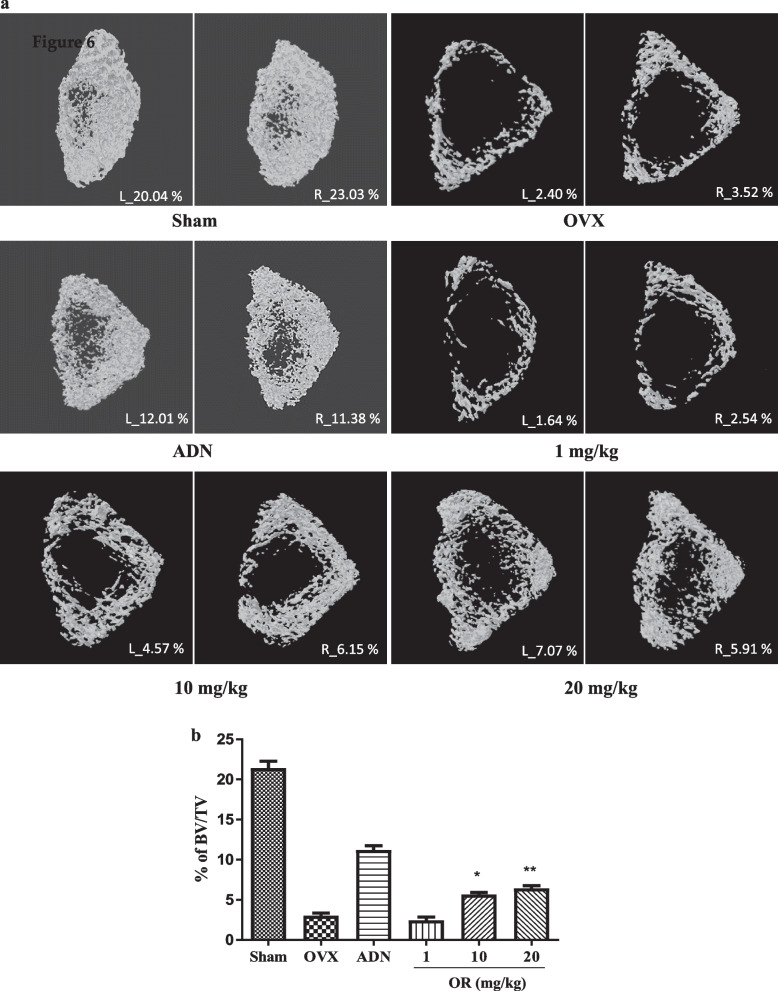
Micro-CT analysis of the femurs in oxyresveratrol (OR)-administered rats. The femurs were analyzed after oral administration of the OR to rats of each group for 8 weeks. (**a**) It was confirmed that bone density was significantly lower in the OVX group, and an increase in bone density was observed at 10 and 20 mg/kg (**b**) Bone volume per total volume(BV/TV) result obtained by oral administration of OR. Data represent the mean ± SEM of experiments, n = 5; *p < 0.05, **p < 0.01 compared with the OVX group. Error bars indicate standard deviation. BV/TV; trabecular separation

Using X-ray microtomography, three-and two-dimensional longitudinal sections were obtained, and the BV/TV was calculated by dividing the femur into left and right sides (Fig. 6a). The BV/TV values in the sham, OVX, and ADN groups were 20.04% and 23.03%, 2.4% and 3.52%, and 12.01% and 11.38%, respectively. OR at 1, 10, and 20 mg/kg resulted in BV/TV values of 1.64% and 2.54%, 4.57% and 6.15%, and 7.07% and 5.91%, respectively. OR at 10 and 20 mg/kg resulted in a mean increase of 2.4% compared to OVX (Fig. 6b).

## Discussion

Although bisphosphonate is often used to treat osteoporosis, excessive suppression of bone resorption could decrease bone turnover, resulting in side effects such as maxillary necrosis [4]. Therefore, there is a need for new osteoporosis medications. However, the effects of most functional substances on osteoporosis or other bone diseases are unclear. Stilbene is a type of plant polyphenols and its derivatives are promising for drug research and development due to its therapeutic application potential [34]. We evaluated the effect of OR, which is reportedly more effective than polyphenol resveratrol, on bone differentiation [19]. Many studies have reported the potential of various natural products for treating osteoporosis [35]. Our results confirm that, at low concentrations, OR exhibits a preventive effect for osteoporosis compared to other natural products.

RANKL, an osteoclastogenic factor, induces phosphorylation of MAPKs (ERK, p38, and JNK), and is important in osteoclast differentiation [36]. RANKL has biphasic activity, causing immediate (5–20 min) and delayed (8–24 h) phosphorylation of MAPKs [36]. RANKL-induced MAPK signal transduction modulates the proliferation of osteoclast precursors and differentiation of osteoclasts [37]. Regarding the role of ERK in osteoclast proliferation, RANKL and RANK binding stimulates tumor necrosis factor receptor-associated factor 6 (TRAF6) and activates ERK, thereby upregulating NFATc1 [36]. Thus, the RANKL/RARNK/TRAF6/ERK cascade leads to the formation of osteoclast precursors and regulates their functions [38, 39]. JNK and p38, activated by the RANKL-RANK signaling cascade, mediate osteoclast apoptosis and promote the differentiation and function of osteoclasts [10, 40–42]. The JNK pathway is necessary up to the osteoclast commitment stage [16], whereas the p38 pathway is important in osteoclast formation and maturation, as well as in bone remodeling and resorption [9, 43]. Deficiency of p38α increases bone mass in young mice with reduced bone resorption [43]. Phosphorylation of p38 promotes the formation of osteoclasts via NF-κB signaling and NFATc1 activation [44, 45]. In this study, 100 nM, 1 μM, and 10 μM OR suppressed the phosphorylation of MAPK factors that promote osteoclast differentiation. Differentiation of osteoclasts involves the commitment, differentiation, fusion, and resorption stages [46]. OR suppressed the expression levels of NFATc1, a marker of the differentiation stage, and of cathepsin K and TRAP, markers of the resorption stage, in a dose-dependent manner. Also, at the molecular level, OR inhibited the expression of MAPK proteins such as ERK, P38, and JNK, and inhibited the expression of NFATc1 target proteins including TRAP, cathepsin K. These findings support the therapeutic potential of OR for diseases related to bone resorption. In this study, it was confirmed that the OR treatment inhibited bone resorption in vivo using an ovariectomized animal model. Ovariectomy has been shown to induce osteopenia with an increase in bone turnover dominating bone resorption. Enhancement of bone resorption results in increases in collagen degradation products. We measured urinary excretion of bone resorption marker, the collagen breakdown product DPD. The effect of OR was confirmed to inhibit DPD, a collagen degradation product. Also, tartrate-resistant acid phosphatase (TRAP) activity is considered an important marker of osteoclasts. Serum concentration is used as a biochemical indicator of osteoclast function and bone resorption. It was confirmed that the serum concentration of TRAP activity was also reduced as much as ADN. Bone mineral density was measured using the ovariectomized femur, and it was confirmed that bone mineral density increased during OR treatment.

In summary, OR at non-cytotoxic concentrations decreased bone resorption, TRAP activity, and actin formation in vitro. Also in vivo, OR decreased the RANKL-induced phosphorylation of MAPK factors and suppressed the levels of marker proteins in the differentiation stage. The effects of ORs on the molecular mechanisms underlying osteoclast differentiation and osteoclastogenesis remain to be elucidated. Our results show that inhibition of osteoclast differentiation and its function in the OR has a potential effect for preventing bone loss. Our study results demonstrated that in osteoclast differentiation induced by RANKL, OR treatment had an anti-osteoclastogenesis effect through an inhibitory mechanism of MAPKs phosphorylation. We have identified OR's effect on osteoclast differentiation in Raw 264.7 cells, which is currently unknown. Future studies should identify OR's effect in rat bone marrow-derived macrophages (BMM) to compare the two results.

## Conclusions

In summary, OR exerted an anti-osteoclastogenic effect by suppressing MAPK phosphorylation. These findings support the therapeutic potential of OR for diseases related to bone resorption. In an ovariectomized animal model, an increase in bone density was observed, although the effect of OR was less than that of alendronate. Further studies are needed to elucidate the mechanism by which OR inhibits osteoclast resorption. Further studies using OR will provide insight into the pathogenesis of osteoporosis.

## Data Availability

All relevant data are contained within the article.

## Abbreviations

OR: Oxyresveratrol
TRAP: Tartrate-resistant acid phosphatase
DPD: Deoxypyridinoline
RANKL: Inhibited receptor activator of nuclear factor kappa-Β ligand
MAPK: Mitogen-activated protein kinases
JNK: C-Jun N-terminal kinase
ERK: Extracellular signal control key
NFATc1: Nuclear factor of activated T-cells, cytoplasmic 1
TRAF6: Tumor necrosis factor receptor-associated factor 6
NF-κB: Nuclear factor-kappa B
M-CSF: Macrophage colony stimulating factor
DC-STAMP: Dendrocyte expressed seven transmembrane protein
DMEM: Dulbecco’s modified Eagle medium
Alpha-MEM: Alpha-Minimum Essential Medium
PBS: Phosphate-buffered saline
ROCs: Round osteoclasts
PCR: Polymerase chain reaction
OVX: Ovariectomy
BV/TV: Bone volume per total volume
AND: Alendronate
